# Supplementary material for: Physiological and Transcriptomic Analysis Revealed the Molecular Mechanism of Pinus koraiensis Responses to Light
Source: Int J Mol Sci. 2022 Nov 6;23(21):13608. doi: 10.3390/ijms232113608 (PMC9653891; doi:10.3390/ijms232113608)
Supplement: Supplementary file 1 [file ijms-23-13608-s001.zip › Supplementary Figures.pdf]

A

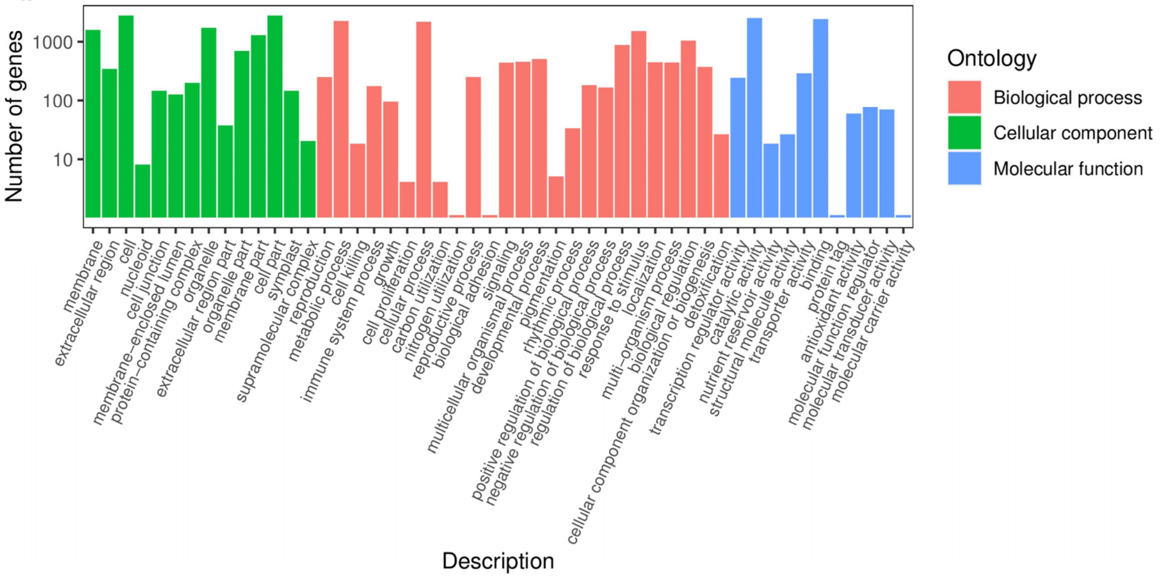
Number of genes
1000
100
10
membrane
extracellular region
cell
nucleoid
cell junction
membrane−enclosed lumen
protein−containing complex
organelle
extracellular region part
organelle part
membrane part
cell part
symplast
supramolecular complex
reproduction
metabolic process
cell killing
immune system process
growth
cell proliferation
cellular process
carbon utilization
nitrogen utilization
reproductive process
biological adhesion
signaling
multicellular organismal process
developmental process
pigmentation
rhythmic process
positive regulation of biological process
negative regulation of biological process
regulation of biological process
response to stimulus
localization
multi−organism process
biological regulation
cellular component organization or biogenesis
detoxification
transcription regulator activity
catalytic activity
nutrient reservoir activity
structural molecule activity
transporter activity
binding
protein tag
antioxidant activity
molecular function regulator
molecular transducer activity
molecular carrier activity
Description
Ontology
Biological process
Cellular component
Molecular function

B

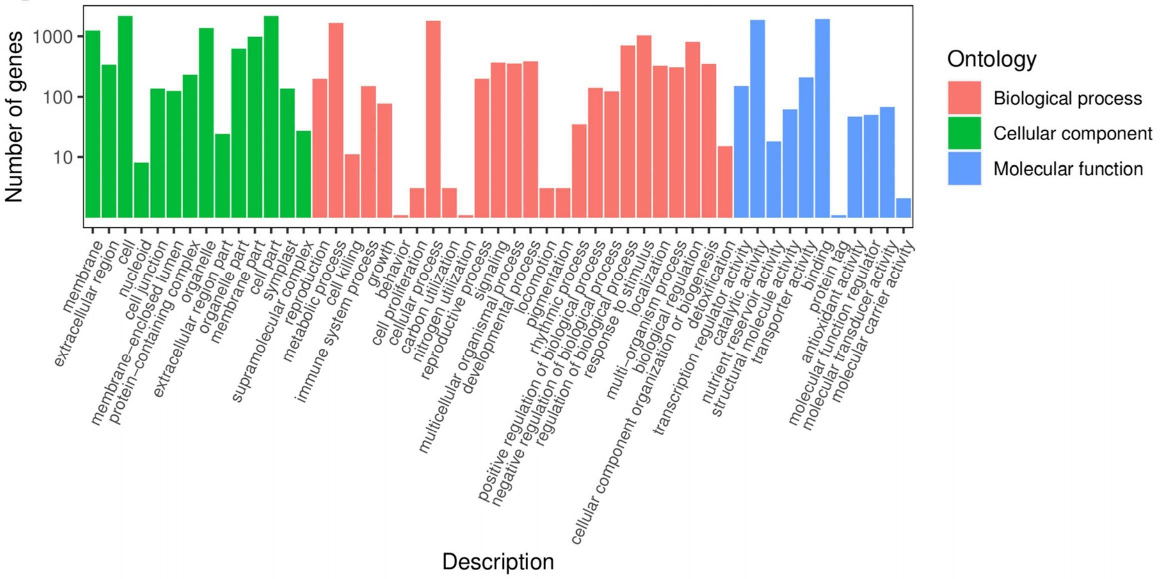
Number of genes
1000
100
10
membrane
extracellular region
cell
nucleoid
cell junction
membrane−enclosed lumen
protein−containing complex
organelle
extracellular region part
organelle part
membrane part
cell part
symplast
supramolecular complex
reproduction
metabolic process
cell killing
immune system process
growth
behavior
cell proliferation
cellular process
carbon utilization
nitrogen utilization
reproductive process
signaling
multicellular organismal process
developmental process
locomotion
pigmentation
rhythmic process
positive regulation of biological process
negative regulation of biological process
regulation of biological process
response to stimulus
localization
multi−organism process
biological regulation
cellular component organization or biogenesis
detoxification
transcription regulator activity
catalytic activity
nutrient reservoir activity
structural molecule activity
transporter activity
binding
protein tag
antioxidant activity
molecular function regulator
molecular transducer activity
molecular carrier activity
Description
Ontology
Biological process
Cellular component
Molecular function

C

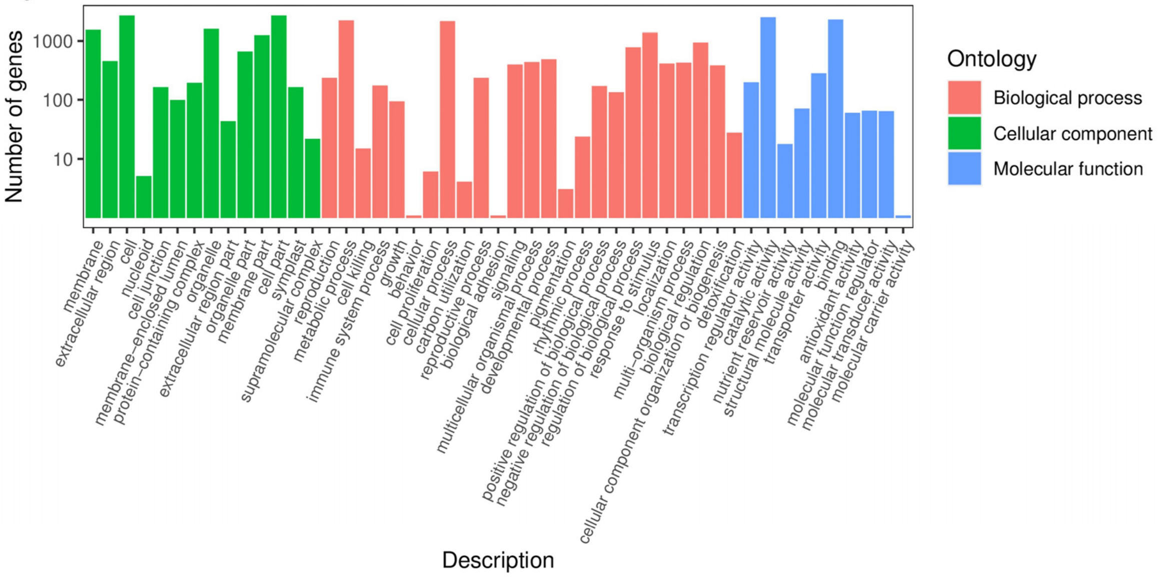
Number of genes
1000
100
10
membrane
extracellular region
cell
nucleoid
cell junction
membrane−enclosed lumen
protein−containing complex
organelle
extracellular region part
organelle part
membrane part
cell part
symplast
supramolecular complex
reproduction
metabolic process
cell killing
immune system process
growth
behavior
cell proliferation
cellular process
carbon utilization
reproductive process
biological adhesion
signaling
multicellular organismal process
developmental process
pigmentation
rhythmic process
positive regulation of biological process
negative regulation of biological process
regulation of biological process
response to stimulus
localization
multi−organism process
biological regulation
cellular component organization or biogenesis
detoxification
transcription regulator activity
catalytic activity
nutrient reservoir activity
structural molecule activity
transporter activity
binding
antioxidant activity
molecular function regulator
molecular transducer activity
molecular carrier activity
Description
Ontology
Biological process
Cellular component
Molecular function

Figure S1: GO classification of differentially expressed genes in *P. koraiensis* under different supplementary lights. (A) The GO classification of DEGs in CK vs. L4. (B) The GO classification of DEGs in CK vs. In4. (C) The GO classification of DEGs in L4 vs. In4.

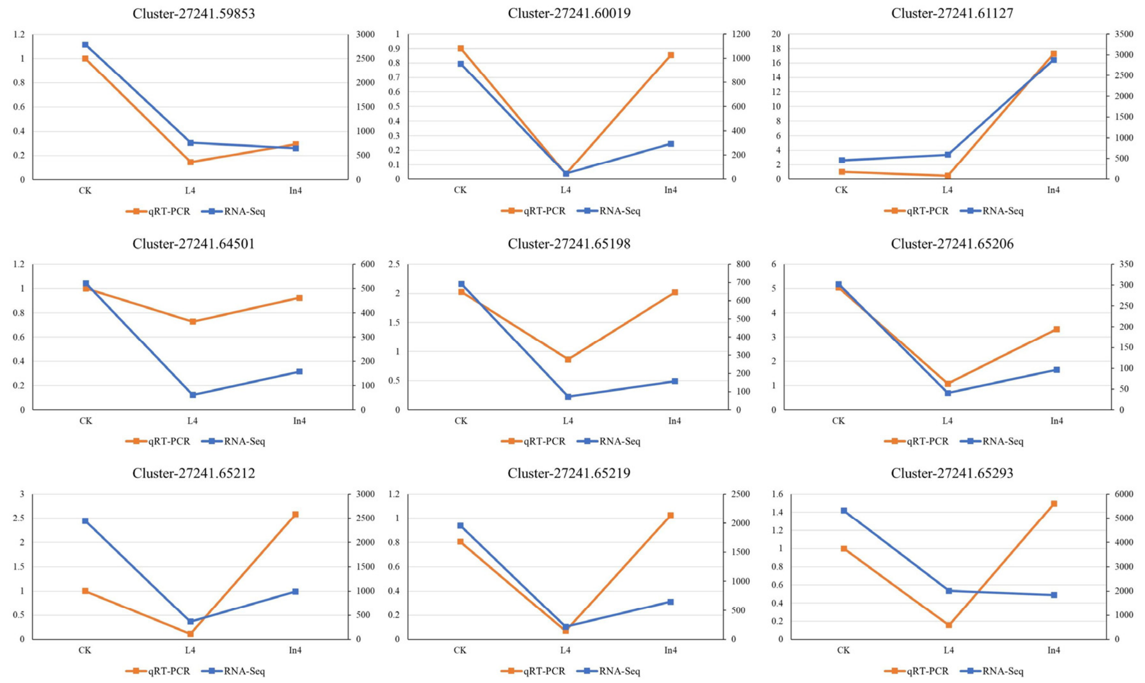

Figure S2: qRT–PCR verification of the expression levels of nine DEGs identified by RNA-Seq. The X-axis represents the different samples. The Y-axis on the right represents the FPKM value obtained by RNA-seq, while the Y-axis on the left indicates the relative gene expression levels analyzed by qRT–PCR.
